# Supplementary material for: Interactions between two functionally distinct aquatic invertebrate herbivores complicate ecosystem- and population-level resilience
Source: PeerJ. 2022 Oct 7;10:e14103. doi: 10.7717/peerj.14103 (PMC9549887; doi:10.7717/peerj.14103)
Supplement: Supplemental Information 1 [file peerj-10-14103-s001.pdf]

SUPPLEMENT TO: Aquatic ecosystem and population level resilience complicated by interactions between invertebrate herbivores

Authors: Jo A. Werba, Alexander C. Phong, Lakhdeep Brar, Acacia Frempong-Manso, Ofure Vanessa Oware and Jurek Kolasa

The supplement contains algal species information.

Figure 1: This figure shows all algal species that represent greater than 0.5% of the community for the starting (A.), mid-point (B.) and end of the experiment (C.). Point color is herbivore combination. Point shape is perturbation treatment. Points are mean and error bars are standard deviation. (N=10 for all treatments)

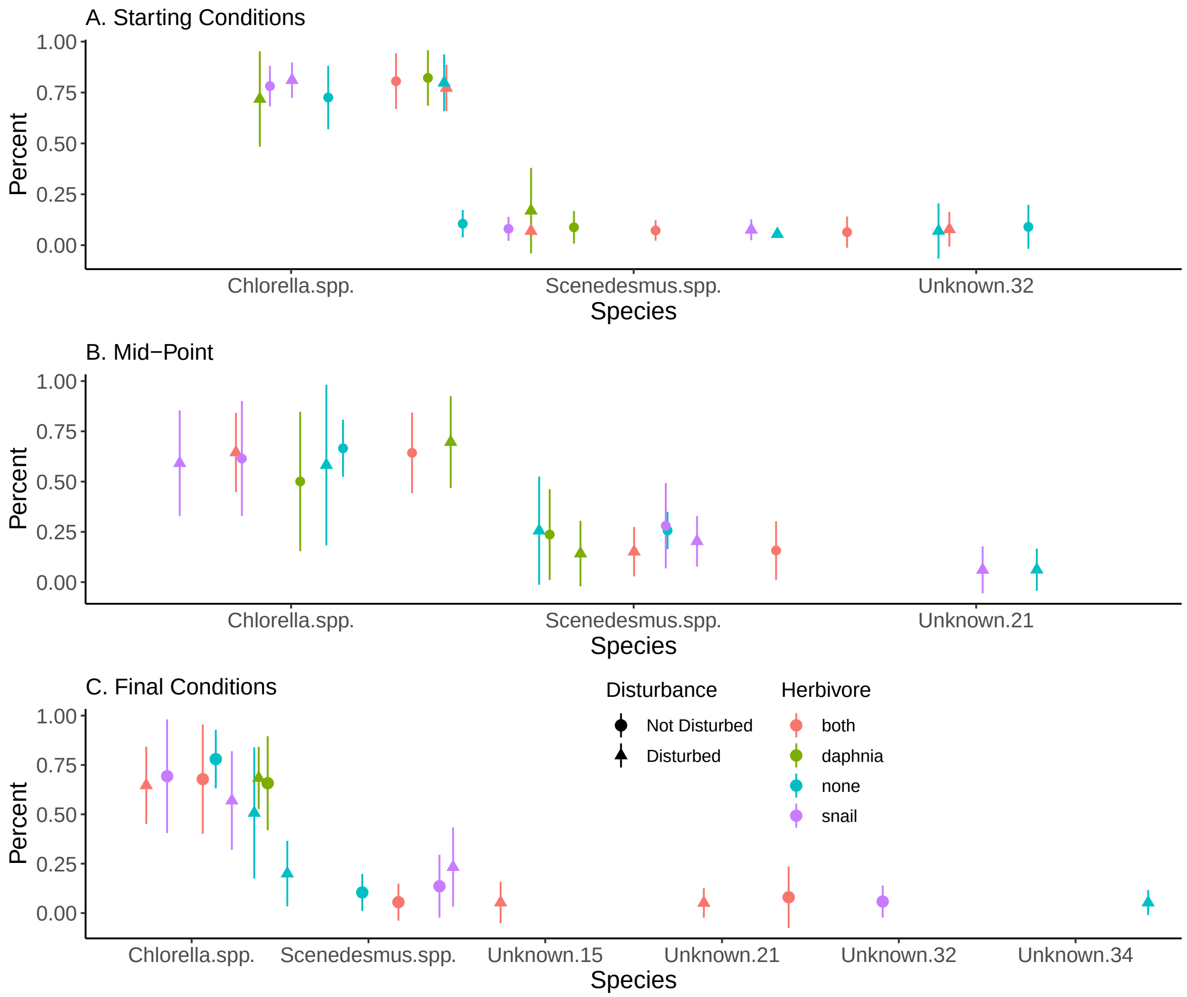

Figure 2: This figure shows all algal species on the first day of the experiment

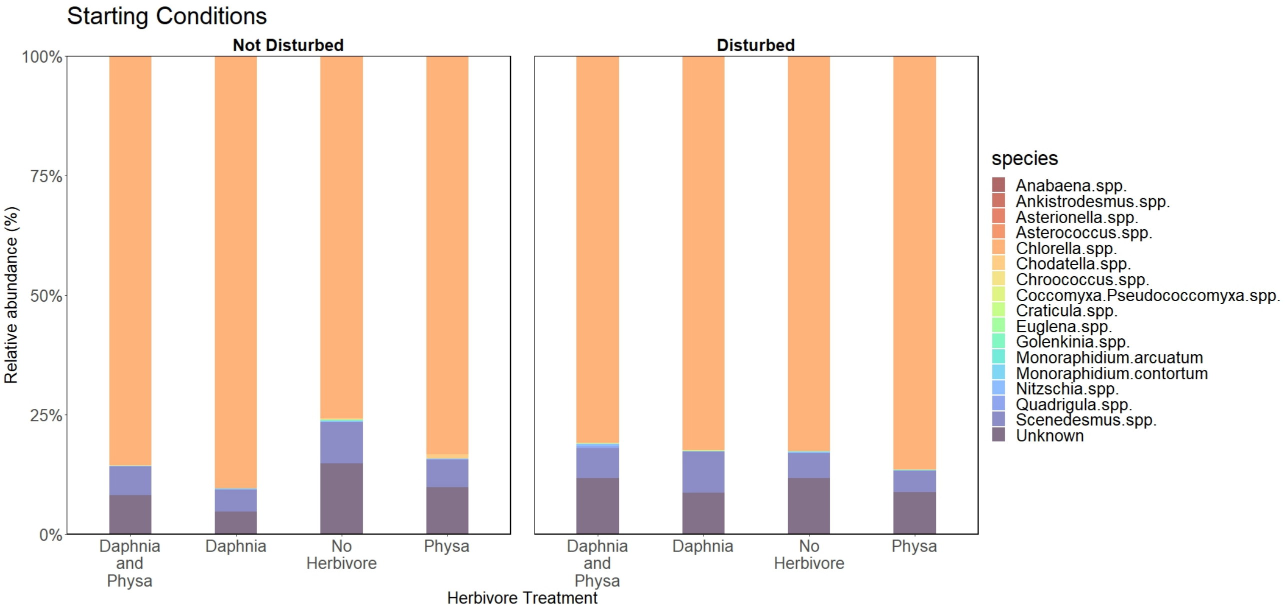

Figure 3: This figure shows all algal species at the mid-point of the experiment prior to any disturbance treatment

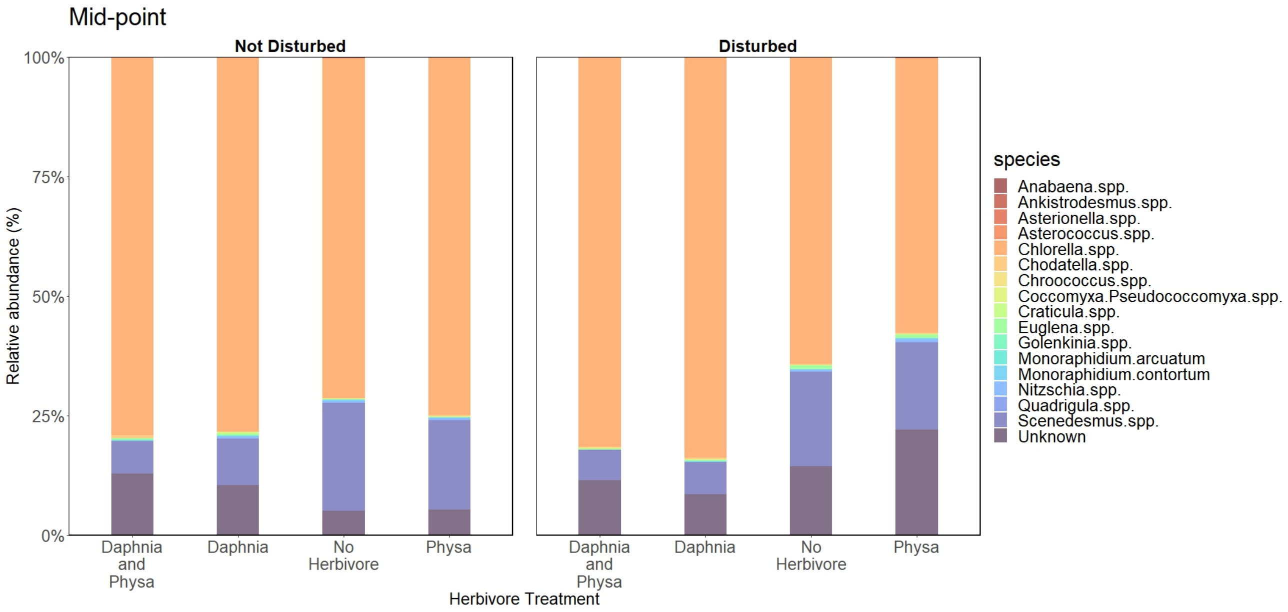

Figure 4: This figure shows all algal species at the end of the experiment

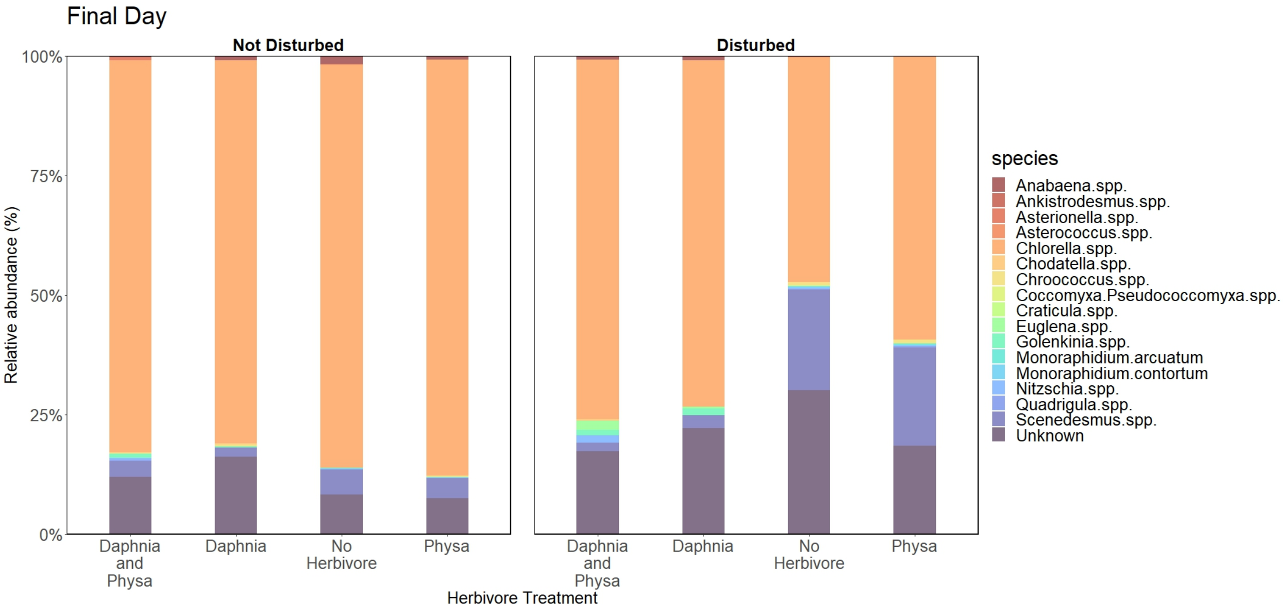

Algae Species Tables

Table 1: Algal species found each treatment

Herbivore Treatment	Disturb	Collection point	Taxa	Count
***D. magna* and *Physa* sp.**	**Not Disturbed**	**1**	Anabaena.spp.	1
			Ankistrodesmus.spp.	2
			Chlorella.spp.	1617
			Chodatella.spp.	1
			Chroococcus.spp.	1
			Nitzschia.spp.	3
			Scenedesmus.spp.	114
			Unknown.36	1
			Unknown.14	2
			Unknown.15	3
			Unknown.17	6
			Unknown.21	3
			Unknown.27	1
			Unknown.30	4
			Unknown.31	6
			Unknown.32	68
			Unknown.34	5
			Unknown.35	1
			Unknown.37	2
			Unknown.38	1
			Unknown.39	19
			Unknown.4	1
			Unknown.41	1
			Unknown.42	2
			Unknown.9	28
		**2**	Chlorella.spp.	718
			Chodatella.spp.	5
			Euglena.spp.	2
			Golenkinia.spp.	3
			Unknown.18	4
			Quadrigula.spp.	1
			Scenedesmus.spp.	61
			Unknown.10	1
			Unknown.11	3
			Unknown.14	1
			Unknown.15	1
			Unknown.17	4
			Unknown.19	1
			Unknown.21	9

Table 1: Algal species found each treatment

Herbivore Treatment	Disturb	Collection point	Taxa	Count
			Unknown.22	5
			Unknown.25	1
			Unknown.26	1
			Unknown.3	2
			Unknown.30	2
			Unknown.32	13
			Unknown.34	5
			Unknown.35	8
			Unknown.37	10
			Unknown.38	4
			Unknown.4	1
			Unknown.41	1
			Unknown.42	3
			Unknown.43	1
			Unknown.44	4
			Unknown.50	5
			Unknown.55	2
			Unknown.57	3
			Unknown.62	1
			Unknown.64	1
			Unknown.65	2
			Unknown.68	3
			Unknown.69	2
			Unknown.70	2
			Unknown.9	11
		**3**	Anabaena.spp.	1
			Asterionella.spp.	2
			Chlorella.spp.	268
			Chroococcus.spp.	1
			Golenkinia.spp.	3
			Unknown.18	3
			Nitzschia.spp.	1
			Quadrigula.spp.	1
			Scenedesmus.spp.	11
			Unknown.14	1
			Unknown.16	1
			Unknown.21	10
			Unknown.23	1
			Unknown.25	1
			Unknown.32	8
			Unknown.35	3
			Unknown.38	2

Table 1: Algal species found each treatment

Herbivore Treatment	Disturb	Collection point	Taxa	Count
			Unknown.41	1
			Unknown.42	1
			Unknown.43	1
			Unknown.44	1
			Unknown.54	1
			Unknown.9	4
***D. magna* and *Physa* sp.**	**Disturbed**	**1**	Ankistrodesmus.spp.	1
			Chlorella.spp.	1064
			Chodatella.spp.	1
			Euglena.spp.	1
			Golenkinia.spp.	1
			Nitzschia.spp.	7
			Quadrigula.spp.	4
			Scenedesmus.spp.	82
			Unknown.14	1
			Unknown.15	1
			Unknown.16	1
			Unknown.17	4
			Unknown.21	5
			Unknown.23	1
			Unknown.25	1
			Unknown.31	8
			Unknown.32	72
			Unknown.34	6
			Unknown.35	5
			Unknown.37	1
			Unknown.38	10
			Unknown.39	12
			Unknown.4	2
			Unknown.42	2
			Unknown.44	6
			Unknown.45	2
			Unknown.48	1
			Unknown.8	1
			Unknown.9	13
		**2**	Chlorella.spp.	608
			Chodatella.spp.	1
			Chroococcus.spp.	2
			Euglena.spp.	2
			known.18	1
			Scenedesmus.spp.	47

Table 1: Algal species found each treatment

Herbivore Treatment	Disturb	Collection point	Taxa	Count
			Unknown.10	2
			Unknown.11	1
			Unknown.14	4
			Unknown.15	1
			Unknown.19	7
			Unknown.21	15
			Unknown.22	3
			Unknown.32	7
			Unknown.34	1
			Unknown.35	3
			Unknown.37	6
			Unknown.38	2
			Unknown.39	1
			Unknown.4	1
			Unknown.41	1
			Unknown.42	1
			Unknown.49	1
			Unknown.50	6
			Unknown.55	2
			Unknown.57	2
			Unknown.60	1
			Unknown.61	1
			Unknown.62	1
			Unknown.64	2
			Unknown.65	1
			Unknown.66	1
			Unknown.68	2
			Unknown.9	9
		**3**	Anabaena.spp.	2
			Chlorella.spp.	200
			Chodatella.spp.	1
			Euglena.spp.	5
			Golenkinia.spp.	3
			Nitzschia.spp.	4
			Scenedesmus.spp.	5
			Unknown.14	1
			Unknown.15	5
			Unknown.16	1
			Unknown.20	1
			Unknown.21	8
			Unknown.22	1
			Unknown.25	1

Table 1: Algal species found each treatment

Herbivore Treatment	Disturb	Collection point	Taxa	Count
			Unknown.26	1
			Unknown.32	12
			Unknown.34	1
			Unknown.35	2
			Unknown.37	1
			Unknown.38	1
			Unknown.39	1
			Unknown.41	1
			Unknown.50	1
			Unknown.56	1
			Unknown.58	1
			Unknown.9	5
***D. magna* only**	**Not Disturbed**	**1**	Ankistrodesmus.spp.	2
			Chlorella.spp.	2180
			Chodatella.spp.	1
			Chroococcus.spp.	1
			Monoraphidium contortum	1
			Nitzschia.spp.	4
			Scenedesmus.spp.	110
			Unknown.10	1
			Unknown.13	1
			Unknown.14	1
			Unknown.15	1
			Unknown.16	4
			Unknown.17	9
			Unknown.21	3
			Unknown.23	1
			Unknown.3	2
			Unknown.31	3
			Unknown.32	32
			Unknown.34	8
			Unknown.35	3
			Unknown.37	2
			Unknown.38	3
			Unknown.39	7
			Unknown.4	1
			Unknown.41	1
			Unknown.43	2
			Unknown.44	5
			Unknown.48	1

Table 1: Algal species found each treatment

Herbivore Treatment	Disturb	Collection point	Taxa	Count
			Unknown.6	1
			Unknown.9	22
		**2**	Chlorella.spp.	559
			Chodatella.spp.	1
			Coccomyxa Pseudococcomyxa.spp.	1
			Craticula.spp.	2
			Euglena.spp.	2
			Golenkinia.spp.	1
			Unknown.18	1
			Monoraphidium arcuatum	1
			Nitzschia.spp.	2
			Quadrigula.spp.	1
			Scenedesmus.spp.	69
			Unknown.10	2
			Unknown.14	1
			Unknown.21	11
			Unknown.22	5
			Unknown.25	2
			Unknown.3	1
			Unknown.30	1
			Unknown.32	14
			Unknown.34	1
			Unknown.35	3
			Unknown.37	12
			Unknown.38	3
			Unknown.4	1
			Unknown.50	5
			Unknown.55	1
			Unknown.56	2
			Unknown.6	1
			Unknown.63	1
			Unknown.68	2
			Unknown.69	1
			Unknown.9	4
		**3**	Anabaena.spp.	3
			Chlorella.spp.	253
			Chodatella.spp.	1
			Chroococcus.spp.	1
			Golenkinia.spp.	1
			Unknown.18	3

Table 1: Algal species found each treatment

Herbivore Treatment	Disturb	Collection point	Taxa	Count
			Scenedesmus.spp.	6
			Unknown.10	1
			Unknown.11	1
			Unknown.14	2
			Unknown.15	5
			Unknown.17	1
			Unknown.19	1
			Unknown.20	1
			Unknown.21	8
			Unknown.22	2
			Unknown.25	1
			Unknown.3	1
			Unknown.31	1
			Unknown.32	15
			Unknown.35	2
			Unknown.38	1
			Unknown.44	1
			Unknown.48	1
			Unknown.63	1
			Unknown.9	2
***D. magna* only**	**Disturbed**	**1**	Anabaena.spp.	1
			Chlorella.spp.	1079
			Chodatella.spp.	1
			Chroococcus.spp.	1
			Euglena.spp.	1
			Golenkinia.spp.	1
			Quadrigula.spp.	1
			Scenedesmus.spp.	113
			Unknown.10	1
			Unknown.14	1
			Unknown.15	6
			Unknown.16	2
			Unknown.17	5
			Unknown.21	1
			Unknown.25	1
			Unknown.27	1
			Unknown.30	4
			Unknown.31	1
			Unknown.32	21
			Unknown.38	24
			Unknown.39	4

Table 1: Algal species found each treatment

Herbivore Treatment	Disturb	Collection point	Taxa	Count
			Unknown.41	2
			Unknown.44	5
			Unknown.45	2
			Unknown.9	32
		**2**	Ankistrodesmus.spp.	1
			Chlorella.spp.	961
			Chodatella.spp.	3
			Chroococcus.spp.	1
			Craticula.spp.	1
			Euglena.spp.	2
			Golenkinia.spp.	1
			Unknown.18	1
			Monoraphidium contortum	1
			Nitzschia.spp.	1
			Scenedesmus.spp.	77
			Unknown.36	1
			Unknown.10	2
			Unknown.11	2
			Unknown.14	1
			Unknown.15	1
			Unknown.17	4
			Unknown.19	2
			Unknown.21	18
			Unknown.22	1
			Unknown.25	2
			Unknown.26	1
			Unknown.31	2
			Unknown.32	7
			Unknown.34	4
			Unknown.35	4
			Unknown.37	8
			Unknown.38	2
			Unknown.4	3
			Unknown.41	1
			Unknown.42	3
			Unknown.44	4
			Unknown.49	1
			Unknown.50	7
			Unknown.52	1
			Unknown.55	1
			Unknown.56	2

Table 1: Algal species found each treatment

Herbivore Treatment	Disturb	Collection point	Taxa	Count
			Unknown.58	1
			Unknown.63	1
			Unknown.65	1
			Unknown.66	1
			Unknown.68	1
			Unknown.9	7
		**3**	Anabaena.spp.	2
			Chlorella.spp.	160
			Euglena.spp.	1
			Golenkinia.spp.	3
			Unknown.18	3
			Scenedesmus.spp.	6
			Unknown.15	4
			Unknown.16	1
			Unknown.17	1
			Unknown.19	1
			Unknown.21	4
			Unknown.22	4
			Unknown.23	2
			Unknown.25	2
			Unknown.27	1
			Unknown.32	10
			Unknown.34	1
			Unknown.35	1
			Unknown.38	5
			Unknown.56	1
			Unknown.60	1
			Unknown.62	1
			Unknown.65	1
			Unknown.9	5
***Physa* sp. only**	**Not Disturbed**	**1**	Ankistrodesmus.spp.	1
			Chlorella.spp.	821
			Chodatella.spp.	8
			Chroococcus.spp.	1
			Nitzschia.spp.	1
			Quadrigula.spp.	1
			Scenedesmus.spp.	57
			Unknown.36	2
			Unknown.10	1
			Unknown.14	2
			Unknown.15	2

Table 1: Algal species found each treatment

Herbivore Treatment	Disturb	Collection point	Taxa	Count
			Unknown.17	2
			Unknown.21	8
			Unknown.31	2
			Unknown.32	22
			Unknown.34	1
			Unknown.35	2
			Unknown.38	5
			Unknown.39	7
			Unknown.42	2
			Unknown.44	3
			Unknown.45	1
			Unknown.48	1
			Unknown.9	34
		**2**	Ankistrodesmus.spp.	3
			Chlorella.spp.	1555
			Chodatella.spp.	4
			Chroococcus.spp.	1
			Coccomyxa Pseudococcomyxa.spp.	1
			Craticula.spp.	1
			Euglena.spp.	2
			Golenkinia.spp.	1
			Unknown.18	2
			Monoraphidium contortum	3
			Nitzschia.spp.	8
			Quadrigula.spp.	2
			Scenedesmus.spp.	388
			Unknown.10	2
			Unknown.14	1
			Unknown.17	14
			Unknown.19	1
			Unknown.21	2
			Unknown.22	4
			Unknown.25	3
			Unknown.26	7
			Unknown.3	1
			Unknown.30	2
			Unknown.31	1
			Unknown.32	6
			Unknown.34	1
			Unknown.35	12

Table 1: Algal species found each treatment

Herbivore Treatment	Disturb	Collection point	Taxa	Count
			Unknown.38	7
			Unknown.42	1
			Unknown.43	4
			Unknown.44	2
			Unknown.45	1
			Unknown.50	6
			Unknown.54	1
			Unknown.55	2
			Unknown.56	1
			Unknown.57	3
			Unknown.61	1
			Unknown.62	4
			Unknown.66	2
			Unknown.67	1
			Unknown.9	15
		**3**	Anabaena.spp.	41
			Ankistrodesmus.spp.	2
			Chlorella.spp.	4429
			Chodatella.spp.	3
			Chroococcus.spp.	12
			Coccomyxa Pseudococcomyxa.spp.	1
			Golenkinia.spp.	2
			Unknown.18	2
			Monoraphidium contortum	5
			Nitzschia.spp.	4
			Scenedesmus.spp.	217
			Unknown.10	2
			Unknown.14	1
			Unknown.15	3
			Unknown.16	2
			Unknown.21	4
			Unknown.23	2
			Unknown.25	2
			Unknown.26	3
			Unknown.3	1
			Unknown.30	2
			Unknown.32	291
			Unknown.35	2
			Unknown.38	3
			Unknown.41	3

Table 1: Algal species found each treatment

Herbivore Treatment	Disturb	Collection point	Taxa	Count
			Unknown.42	1
			Unknown.43	5
			Unknown.45	8
			Unknown.48	2
			Unknown.50	5
			Unknown.52	1
			Unknown.54	1
			Unknown.60	4
			Unknown.61	1
			Unknown.62	3
			Unknown.63	3
			Unknown.64	10
			Unknown.67	1
			Unknown.9	12
***Physa* sp. only**	**Disturbed**	**1**	Ankistrodesmus.spp.	2
			Chlorella.spp.	1374
			Chodatella.spp.	1
			Euglena.spp.	1
			Golenkinia.spp.	1
			Nitzschia.spp.	1
			Quadrigula.spp.	1
			Scenedesmus.spp.	70
			Unknown.10	1
			Unknown.11	1
			Unknown.14	4
			Unknown.16	1
			Unknown.17	10
			Unknown.21	13
			Unknown.27	2
			Unknown.30	1
			Unknown.31	4
			Unknown.32	8
			Unknown.34	2
			Unknown.35	2
			Unknown.38	4
			Unknown.39	18
			Unknown.4	2
			Unknown.42	10
			Unknown.44	10
			Unknown.45	2
			Unknown.9	45

Table 1: Algal species found each treatment

Herbivore Treatment	Disturb	Collection point	Taxa	Count
		**2**	Ankistrodesmus.spp.	7
			Chlorella.spp.	1160
			Chodatella.spp.	5
			Chroococcus.spp.	1
			Coccomyxa Pseudococcomyxa.spp.	1
			Craticula.spp.	1
			Euglena.spp.	15
			Golenkinia.spp.	1
			known.18	3
			Monoraphidium contortum	5
			Nitzschia.spp.	10
			Quadrigula.spp.	3
			Scenedesmus.spp.	369
			Unkniwn.36	4
			Unknown.10	8
			Unknown.11	4
			Unknown.14	6
			Unknown.15	1
			Unknown.17	6
			Unknown.19	2
			Unknown.21	259
			Unknown.22	11
			Unknown.25	2
			Unknown.26	2
			Unknown.3	2
			Unknown.30	4
			Unknown.31	1
			Unknown.32	4
			Unknown.34	2
			Unknown.35	18
			Unknown.37	4
			Unknown.38	4
			Unknown.39	2
			Unknown.4	2
			Unknown.42	8
			Unknown.43	2
			Unknown.44	8
			Unknown.50	8
			Unknown.55	2
			Unknown.57	4

Table 1: Algal species found each treatment

Herbivore Treatment	Disturb	Collection point	Taxa	Count
			Unknown.6	3
			Unknown.60	1
			Unknown.62	1
			Unknown.65	1
			Unknown.66	23
			Unknown.68	1
			Unknown.69	1
			Unknown.9	31
		**3**	Anabaena.spp.	3
			Ankistrodesmus.spp.	5
			Chlorella.spp.	2686
			Chodatella.spp.	1
			Chroococcus.spp.	36
			Euglena.spp.	2
			Golenkinia.spp.	9
			Unknown.18	12
			Monoraphidium arcuatum	1
			Monoraphidium contortum	10
			Nitzschia.spp.	7
			Quadrigula.spp.	10
			Scenedesmus.spp.	937
			Unknown.36	8
			Unknown.10	33
			Unknown.11	7
			Unknown.14	3
			Unknown.15	7
			Unknown.16	1
			Unknown.17	30
			Unknown.19	3
			Unknown.21	62
			Unknown.22	1
			Unknown.23	4
			Unknown.25	10
			Unknown.26	14
			Unknown.27	6
			Unknown.3	4
			Unknown.30	2
			Unknown.32	153
			Unknown.34	117
			Unknown.35	26

Table 1: Algal species found each treatment

Herbivore Treatment	Disturb	Collection point	Taxa	Count
			Unknown.37	3
			Unknown.38	41
			Unknown.39	9
			Unknown.4	1
			Unknown.41	1
			Unknown.43	17
			Unknown.44	4
			Unknown.45	9
			Unknown.48	25
			Unknown.50	7
			Unknown.51	4
			Unknown.54	18
			Unknown.55	1
			Unknown.56	1
			Unknown.57	6
			Unknown.58	1
			Unknown.60	14
			Unknown.61	2
			Unknown.62	26
			Unknown.63	8
			Unknown.64	6
			Unknown.65	2
			Unknown.9	128
No Herbivore	**Not Disturbed**	**1**	Ankistrodesmus.spp.	1
			Chlorella.spp.	969
			Chodatella.spp.	2
			Chroococcus.spp.	1
			Euglena.spp.	1
			Golenkinia.spp.	2
			Monoraphidium contortum	1
			Nitzschia.spp.	2
			Scenedesmus.spp.	111
			Unknown.36	3
			Unknown.11	1
			Unknown.14	2
			Unknown.16	3
			Unknown.17	6
			Unknown.21	8
			Unknown.23	1
			Unknown.25	2

Table 1: Algal species found each treatment

Herbivore Treatment	Disturb	Collection point	Taxa	Count
			Unknown.27	1
			Unknown.3	1
			Unknown.30	1
			Unknown.32	103
			Unknown.34	3
			Unknown.35	4
			Unknown.37	2
			Unknown.38	12
			Unknown.39	11
			Unknown.40	1
			Unknown.42	1
			Unknown.43	1
			Unknown.44	6
			Unknown.45	1
			Unknown.50	2
			Unknown.51	1
			Unknown.52	1
			Unknown.9	11
		**2**	Anabaena.spp.	1
			Ankistrodesmus.spp.	4
			Chlorella.spp.	1240
			Chodatella.spp.	2
			Chroococcus.spp.	1
			Craticula.spp.	2
			Euglena.spp.	3
			Unknown.18	1
			Monoraphidium contortum	4
			Nitzschia.spp.	5
			Quadrigula.spp.	1
			Scenedesmus.spp.	395
			Unknown.36	1
			Unknown.10	4
			Unknown.14	1
			Unknown.17	4
			Unknown.19	1
			Unknown.21	3
			Unknown.22	3
			Unknown.25	1
			Unknown.26	3
			Unknown.3	1
			Unknown.30	3

Table 1: Algal species found each treatment

Herbivore Treatment	Disturb	Collection point	Taxa	Count
			Unknown.32	2
			Unknown.34	3
			Unknown.35	2
			Unknown.37	1
			Unknown.38	2
			Unknown.43	3
			Unknown.45	1
			Unknown.50	8
			Unknown.54	1
			Unknown.55	2
			Unknown.56	2
			Unknown.6	1
			Unknown.60	4
			Unknown.62	2
			Unknown.64	4
			Unknown.65	1
			Unknown.66	1
			Unknown.68	1
			Unknown.69	1
			Unknown.9	20
		**3**	Anabaena.spp.	73
			Ankistrodesmus.spp.	5
			Chlorella.spp.	3543
			Chodatella.spp.	4
			Chroococcus.spp.	1
			Golenkinia.spp.	1
			Unknown.18	3
			Monoraphidium contortum	5
			Nitzschia.spp.	6
			Scenedesmus.spp.	224
			Unknown.10	2
			Unknown.11	1
			Unknown.14	2
			Unknown.15	1
			Unknown.17	5
			Unknown.19	1
			Unknown.20	1
			Unknown.21	1
			Unknown.23	1
			Unknown.26	3
			Unknown.3	1

Table 1: Algal species found each treatment

Herbivore Treatment	Disturb	Collection point	Taxa	Count
			Unknown.30	1
			Unknown.31	1
			Unknown.32	271
			Unknown.34	4
			Unknown.35	4
			Unknown.38	2
			Unknown.39	4
			Unknown.4	1
			Unknown.41	2
			Unknown.44	3
			Unknown.45	1
			Unknown.48	1
			Unknown.50	3
			Unknown.51	1
			Unknown.52	2
			Unknown.54	1
			Unknown.55	2
			Unknown.60	4
			Unknown.62	1
			Unknown.9	15
No Herbivore	**Disturbed**	**1**	Chlorella.spp.	1347
			Golenkinia.spp.	1
			Unknown.18	1
			Nitzschia.spp.	4
			Scenedesmus.spp.	87
			Unknown.11	2
			Unknown.13	1
			Unknown.15	7
			Unknown.16	2
			Unknown.17	13
			Unknown.21	9
			Unknown.23	2
			Unknown.25	4
			Unknown.27	2
			Unknown.30	1
			Unknown.31	6
			Unknown.32	76
			Unknown.34	1
			Unknown.35	2
			Unknown.37	2
			Unknown.38	9

Table 1: Algal species found each treatment

Herbivore Treatment	Disturb	Collection point	Taxa	Count
			Unknown.39	11
			Unknown.42	1
			Unknown.44	4
			Unknown.9	34
		**2**	Ankistrodesmus.spp.	5
			Chlorella.spp.	1607
			Chodatella.spp.	5
			Chroococcus.spp.	1
			Craticula.spp.	2
			Euglena.spp.	21
			known.18	5
			Monoraphidium contortum	3
			Nitzschia.spp.	9
			Quadrigula.spp.	1
			Scenedesmus.spp.	494
			Unknown.10	7
			Unknown.11	4
			Unknown.15	1
			Unknown.17	12
			Unknown.19	1
			Unknown.21	207
			Unknown.25	3
			Unknown.26	1
			Unknown.3	1
			Unknown.30	8
			Unknown.31	1
			Unknown.32	5
			Unknown.34	2
			Unknown.35	1
			Unknown.37	5
			Unknown.38	39
			Unknown.41	1
			Unknown.42	2
			Unknown.43	1
			Unknown.44	7
			Unknown.50	16
			Unknown.54	1
			Unknown.60	2
			Unknown.61	1
			Unknown.62	2
			Unknown.66	14

Table 1: Algal species found each treatment

Herbivore Treatment	Disturb	Collection point	Taxa	Count
			Unknown.9	12
		**3**	Anabaena.spp.	6
			Ankistrodesmus.spp.	5
			Chlorella.spp.	1927
			Chodatella.spp.	3
			Chroococcus.spp.	24
			Coccomyxa Pseudococcomyxa.spp.	3
			Euglena.spp.	2
			Golenkinia.spp.	6
			Unknown.18	19
			Monoraphidium contortum	9
			Nitzschia.spp.	11
			Quadrigula.spp.	5
			Scenedesmus.spp.	861
			Unknown.36	6
			Unknown.10	48
			Unknown.11	6
			Unknown.14	4
			Unknown.15	2
			Unknown.16	2
			Unknown.17	108
			Unknown.21	111
			Unknown.22	2
			Unknown.23	4
			Unknown.25	29
			Unknown.26	33
			Unknown.27	1
			Unknown.28	6
			Unknown.3	17
			Unknown.30	1
			Unknown.31	7
			Unknown.32	79
			Unknown.34	281
			Unknown.35	63
			Unknown.37	2
			Unknown.38	70
			Unknown.39	13
			Unknown.4	2
			Unknown.40	1
			Unknown.41	1

Table 1: Algal species found each treatment

Herbivore Treatment	Disturb	Collection point	Taxa	Count
			Unknown.43	9
			Unknown.44	8
			Unknown.45	13
			Unknown.48	75
			Unknown.50	4
			Unknown.52	3
			Unknown.54	3
			Unknown.55	1
			Unknown.56	1
			Unknown.57	14
			Unknown.6	2
			Unknown.60	33
			Unknown.61	1
			Unknown.62	39
			Unknown.63	5
			Unknown.64	25
			Unknown.65	3
			Unknown.66	3
			Unknown.9	73
